# Supplementary material for: The mitochondrial negative regulator MCJ is a therapeutic target for acetaminophen-induced liver injury
Source: Nat Commun. 2017 Dec 12;8:2068. doi: 10.1038/s41467-017-01970-x (PMC5727217; doi:10.1038/s41467-017-01970-x)
Supplement: Supplementary file 1 — Supplementary Information [file 41467_2017_1970_MOESM1_ESM.pdf]

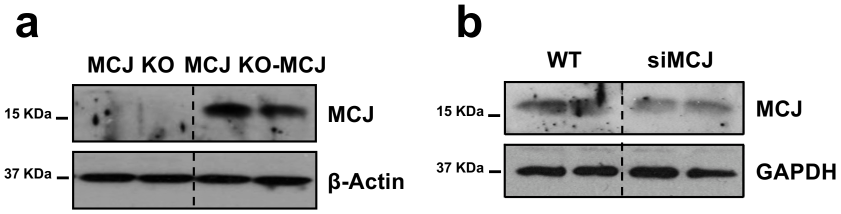

Supplementary Figure 1. MCJ expression determines the susceptibility of hepatocytes to APAP toxicity. (a) MCJ expression was restored in MCJ KO hepatocytes (MCJ KO-MCJ) by transfection with a MCJ-expressing construct as shown by Western blot. (b) MCJ expression was reduced in WT hepatocytes (siMCJ) by transfection with a plasmid containing small interfering RNA sequences targeting MCJ as determined by Western blot.

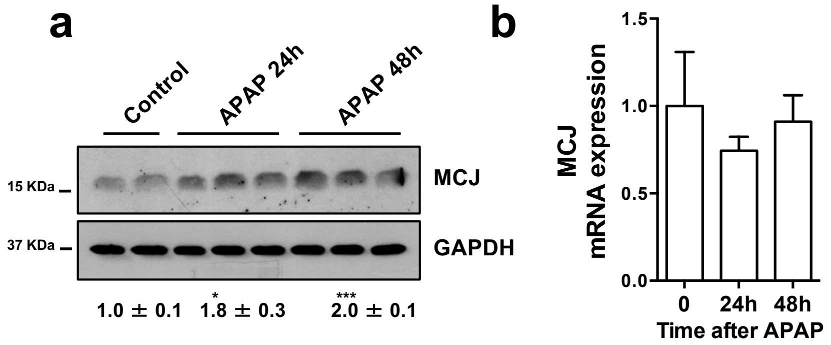

Supplementary Figure 2. MCJ expression in the APAP overdose mouse model. MCJ expression levels in liver in WT mice treated with APAP for 24 and 48h determined by (a) Western blotting and (b) qPCR (n=6). Values are represented as mean ± SEM. *P < 0.05, ***P < 0.001 (APAP *vs* Control).

a

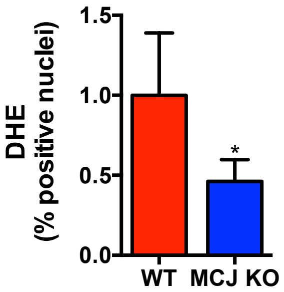

Supplementary Figure 3. MCJ deletion protects against APAP hepatotoxicity *in vivo*. WT and MCJ KO mice (n=6) were treated with APAP 360mg/kg for 6h. (a) ROS *in vivo* measured by DHE staining in liver sections. Values are represented as mean ± SEM. *P < 0.05 (MCJ KO *vs* WT).

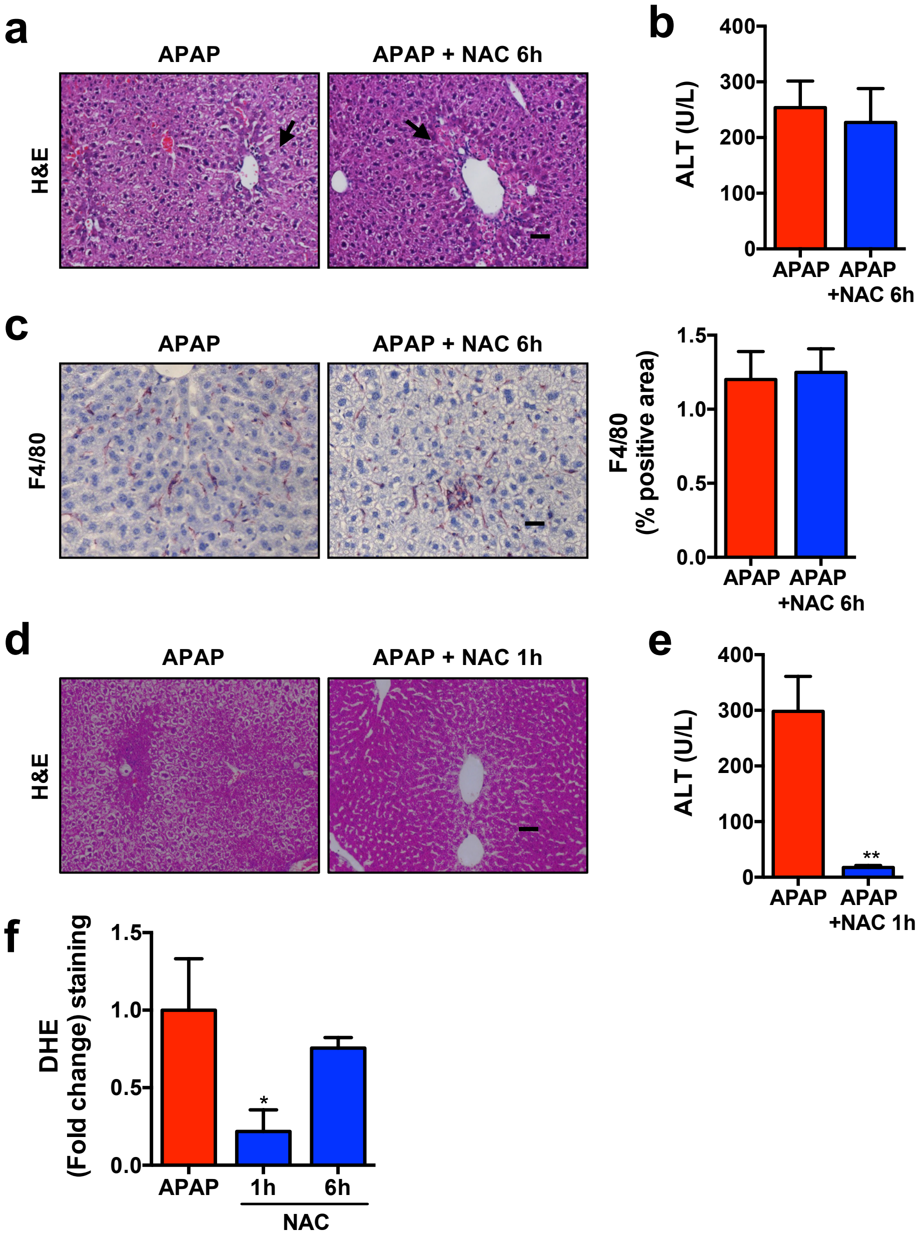

Supplementary Figure 4. NAC shows less effectiveness than siMCJ treatment against APAP-induced liver damage. (a-c) WT mice were treated with APAP 360mg/kg and 6h later, half of the animals (n=4) received NAC 1200mg/kg. (a) Liver necrosis was assessed by H&E staining, (b) serum ALT levels in APAP and APAP+NAC treated animals, (c) liver inflammation was assessed by F4/80 staining. (d and e) WT mice were treated with APAP 360mg/kg and 1h later, half of the animals (n=4) received NAC 1200mg/kg. (d) Liver necrosis was assessed by H&E staining, (e) serum ALT levels in APAP and APAP+NAC treated animals. (f) ROS *in vivo* measured by DHE staining in liver sections of APAP and NAC treated animals. Scale bar corresponds to 100 μm. Values are represented as mean ± SEM. *P < 0.05, **P < 0.01 (APAP+NAC *vs* APAP).

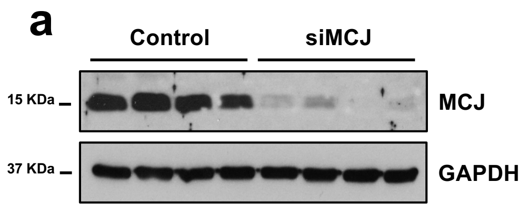

Supplementary Figure 5. . MCJ liver-specific silencing prevents against APAP-induced liver injury *in vivo*. WT mice were treated with APAP 360mg/kg and 24h after control (Control) (n=6) or a MCJ specific siRNA (siMCJ) (n=6) was intravenously injected. (a) MCJ silencing was evaluated by Western blotting.

Uncropped blots for Figure 1C

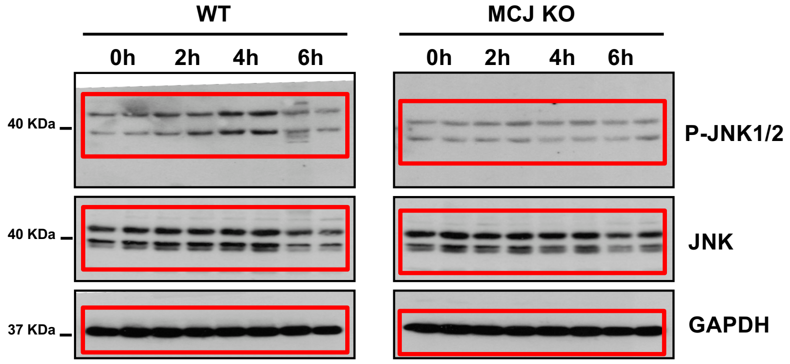

Uncropped blots for Figure 2H

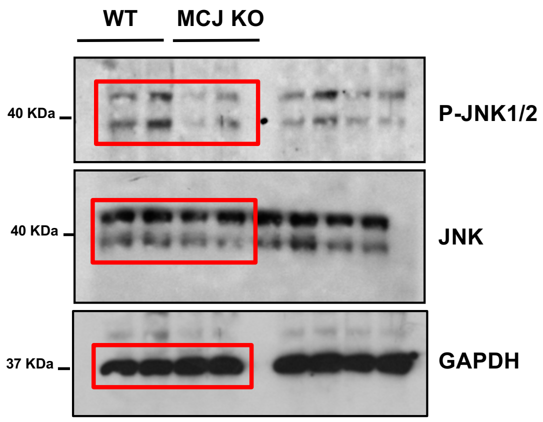

Uncropped blots for Figure 3A

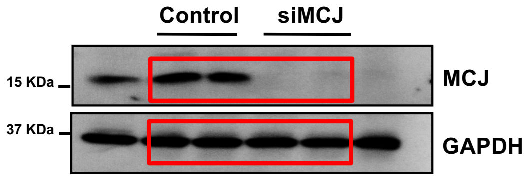

Uncropped blots for Figure 4E

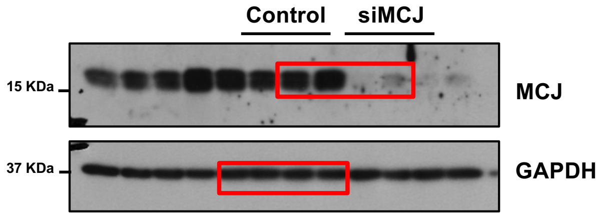

Uncropped blots for Figure 5D

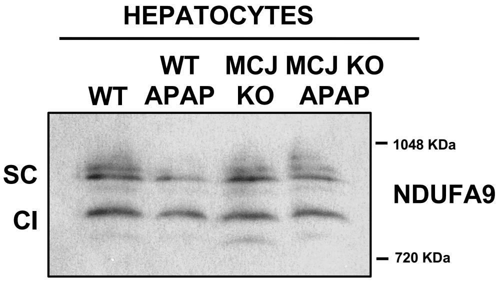

Uncropped blots for Figure 5E

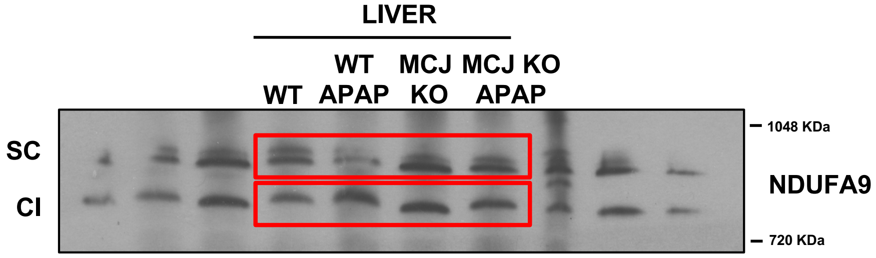

Uncropped blots for Supplementary Figure 1A

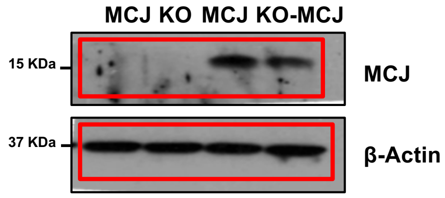

Uncropped blots for Supplementary Figure 1B

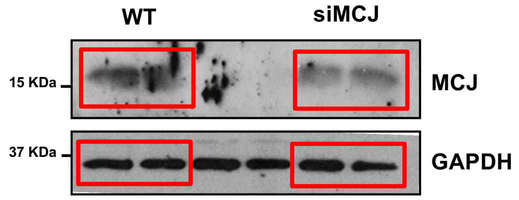

Uncropped blots for Supplementary Figure 2A

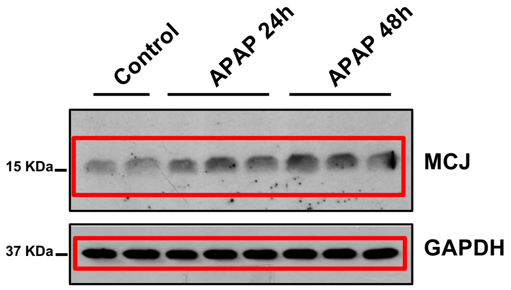

Uncropped blots for Supplementary Figure 5A

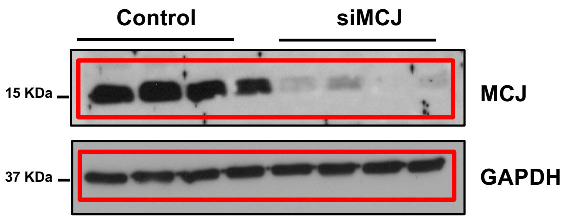

Supplementary Figure 6. **Uncropped blots for figures and supplementary figures.**

Supplementary Table I. Optimal Incubation Conditions, Concentration, Reference and Supplier for Each Specific Antibody Analyzed by Western Blotting

Antibody	Supplier	Catalogue/Clone Nº	Dilution	Incubation solution
GAPDH	Abcam	ab8245	1/10000	TBS-Tween(0,1%)-milk(5%)
JNK1/2 pT183/Y185	Invitrogen	44682G	1/1000	TBS-Tween(0,1%)-milk(5%)
JNK	Cell Signaling	9252S	1/1000	TBS-Tween(0,1%)-milk(5%)
Anti- mouse MCJ	Provided by Rincon, M.*		1/500	TBS-Tween(0,1%)-milk(5%)
NDUFA9	Invitrogen	459100	1/1000	TBS-Tween(0,1%)-milk(5%)

*Rabbit polyclonal antibody against N-terminal of mouse MCJ.

Hatle, K., Gummadidala, P., Navasa, N., Bernardo, E., Dodge, J., Silverstrim, B., Fortner, K., Burg, E., Suratt, B.T., Hammer, J., Radermacher, M., Taatjes, D.J., Thornton, T., Anguita, J., **Rincon, M.,** (2013) "MCJ/DnaJC15, an endogenous mitochondrial repressor of the respiratory chain that controls metabolic alterations". Mol. Cell. Biol. 33, 2302-2314.

Supplementary Table II
Sequence of primers used for RT-qPCR analysis

Gene name	Symbol	Species	Sequence	
Chemokine (C-C motif) ligand 5	*Ccl5*	*Mus musculus*	Forward	5´- CCCTCACCATCATCCTCACT-3´
			Reverse	5´-CCTTCGAGTGACAAACACGA-3´
Chemokine (C-C motif) receptor 5	*Ccr5*	*Mus musculus*	Forward	5´-GTCAGAACGGTCAACTTTGGG-3´
			Reverse	5´-GTGTGGAAAATGAGGACTGCAT-3´
Interleukin 1β	*Il1β*	*Mus musculus*	Forward	5´-GCCACCTTTTGACAGTGATGAG-3´
			Reverse	5´-GACAGCCCAGGTCAAAGGTT-3´
Interleukin 6	*Il6*	*Mus musculus*	Forward	5'-AAGTGAGCAGATAGCACAGTTGG-3'
			Reverse	5'-AATCTTCCTTGCTGTTGCCGTC-3'
Methylation J-controlled protein	*Dnajc15*	*Mus musculus*	Forward	5'-ACGCCGACATCGACCACACAG-3'
			Reverse	5'-AATCTTCCTTGCTGTTGCCGTC-3'
